# Development of a deep learning based image processing tool for enhanced organoid analysis

**DOI:** 10.1038/s41598-023-46485-2

**Published:** 2023-11-13

**Authors:** Taeyun Park, Taeyul K. Kim, Yoon Dae Han, Kyung-A Kim, Hwiyoung Kim, Han Sang Kim

**Affiliations:** 1 Department of Artificial Intelligence, Yonsei University, Seoul, Korea; 2 Department of Internal Medicine, Graduate School of Medical Science, Brain Korea 21 Project, Yonsei University College of Medicine, Seoul, Korea; 3 Department of Surgery, Yonsei University College of Medicine, Seoul, Korea; 4 Department of Biomedical Systems Informatics, Yonsei University College of Medicine, 50 Yonsei-ro, Seodaemun-gu, Seoul, 03722 Republic of Korea; 5 Center for Clinical Imaging Data Science (CCIDS), Yonsei University College of Medicine, Seoul, Korea; 6 Institute for Innovation in Digital Healthcare (IIDH), Yonsei University Health System, Seoul, Korea; 7 Yonsei Cancer Center, Division of Medical Oncology, Department of Internal Medicine, Yonsei University College of Medicine, 50 Yonsei-ro, Seodaemun-gu, Seoul, 03722 Republic of Korea

**Keywords:** Biological techniques, Computational biology and bioinformatics

## Abstract

Contrary to 2D cells, 3D organoid structures are composed of diverse cell types and exhibit morphologies of various sizes. Although researchers frequently monitor morphological changes, analyzing every structure with the naked eye is difficult. Given that deep learning (DL) has been used for 2D cell image segmentation, a trained DL model may assist researchers in organoid image recognition and analysis. In this study, we developed OrgaExtractor, an easy-to-use DL model based on multi-scale U-Net, to perform accurate segmentation of organoids of various sizes. OrgaExtractor achieved an average dice similarity coefficient of 0.853 from a post-processed output, which was finalized with noise removal. Correlation between CellTiter-Glo assay results and daily measured organoid images shows that OrgaExtractor can reflect the actual organoid culture conditions. The OrgaExtractor data can be used to determine the best time point for organoid subculture on the bench and to maintain organoids in the long term.

## Introduction

Organoids are 3D structures composed of numerous cells from pluripotent stem cells or adult stem cells of organs^1^. Because diverse cell types are derived from stem cells, organoids mimic human native organs better than traditional 2D culture systems^2^. Organoids have become a precise preclinical model for researching personalized drugs and organ-specific diseases^3,4^. Optimization of the organoid culture conditions requires periodic monitoring and precise interpretation by researchers^2^. Morphological features such as area, perimeter, or eccentricity are variables for the evaluation of the growth of organoids^5^. Cultured organoids have various features, and understanding their morphological heterogeneity is required to effectively handle organoids. As multiple features are comprehensively used to understand morphology, interpreting images of organoids and obtaining structural information present significant challenges.

In the biomedical field, image processing based on artificial intelligence (AI) can enable effective image data analysis^6^. Recently, AI-based image analysis models outperformed human labor in terms of the time consumed and accuracy^7^. Deep learning (DL) is a subset of the field of machine learning (and therefore AI), which imitates knowledge acquisition by humans^8^. DL models convert convoluted digital images into clear and meaningful subjects^9^. The application of DL-based image analysis includes analyzing cell images^10^ and predicting cell measurements^11^, affording scientists an effective interpretation system.

Recently, DL models have been developed for analyzing organoid images, which include MOrgAna^12^ that quantifies and visualizes morphological information in image datasets, and deepOrganoid^13^ that assesses the changes in organoid viability during drug screening. Despite these advancements, more accurate organoid recognition and visualization of general information from a single organoid is still required. Therefore, researchers require an auxiliary tool to comprehend organoid images and assess their culture conditions.

To address these unmet needs, we developed OrgaExtractor a DL-based organoid image analysis algorithm. OrgaExtractor was designed to overcome the current inefficiency in analyzing organoid images. We developed an accurate organoid segmentation algorithm using a few collected organoid images. OrgaExtractor is based on a multi-scale U-Net, successfully performing challenging segmentation of small-size organoids^14^. With simple image processing methods, we can obtain a fine binary mask to analyze various information about organoids shown in the image.

OrgaExtractor is user-friendly and requires minimal image adjustment for researchers unfamiliar with programming. It exhibits > 85% accuracy in recognizing colon organoids from images, thus showing similarity to manual recognition. The measurements extracted by OrgaExtractor accurately reflect the actual organoid culture conditions, as determined by the metrics used. Researchers can select the metrics that best suit their analysis, and the analyzed data can be easily visualized. Using visualized data, researchers can continuously monitor the growth of organoids under specified culture conditions in real time. Using brightfield images without fluorescence staining, estimating the cell numbers of organoids through a non-invasive approach is feasible. Owing to these features, OrgaExtractor can be a valuable tool for researchers who intend to analyze organoid images right on the lab bench. Researchers with minimal or no programming experience can edit the reposited code to extract the required metrics, according to user manuals written on our GitHub repository.

## Results

### Development of OrgaExtractor as a deep learning-based organoid image processing tool

**Figure 1:**
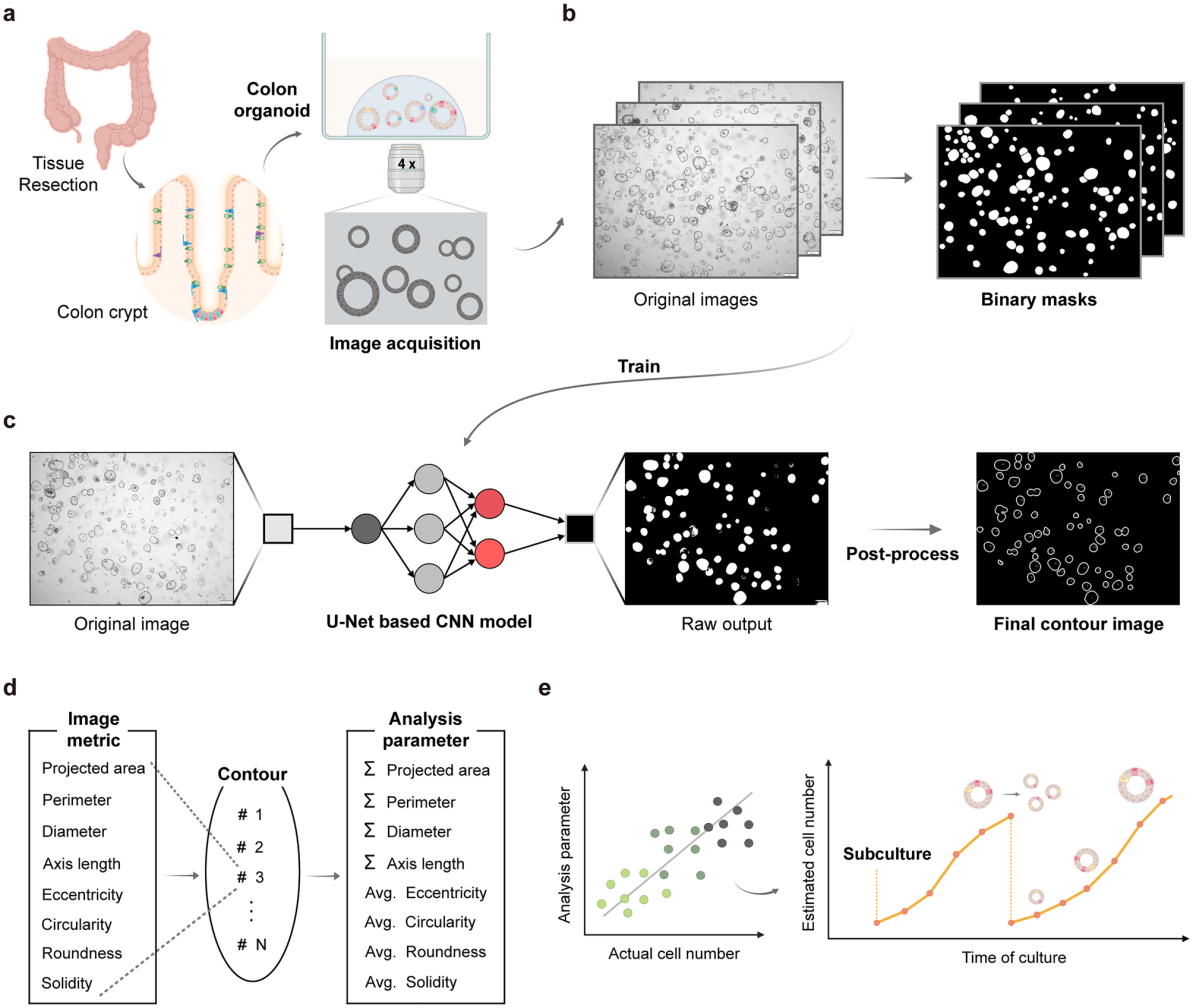
Development workflow of OrgaExtractor and analysis of organoid images. (**a**) Overview of normal colon organoid establishment and organoid image acquisition. See also Supplementary Table S1. (**b**) Pairs of organoid images for training OrgaExtractor: original images and their binary mask images. See also Supplementary Table S2. (**c**) Raw output from the U-Net OrgaExtractor model and the post-processed contour images are shown. See also Supplementary Figs. S1–S2. (**d**) Information regarding each organoid is extracted from the final contour image using OrgaExtractor based on the metrics and parameters used for image analysis. See also Supplementary Table S3. (**e**) Analysis of correlation between image parameters and actual cell numbers. Visualization of the extracted information to evaluate organoid culture conditions.

First, we collected fresh human colon tissues adjacent to the cancer region containing normal colon crypts. Colon crypts were dissociated from tissues and seeded in a gelatinous protein mixture that mimicked the extracellular matrix in human tissue. After a few passages, proliferative adult stem cells in colon crypts were established into patient-derived normal colon organoids (Supplementary Table S1). Although organoids were grown in a 3D culture environment, 2D-projected microscopic images were captured as organoid images in this study (Fig. 1a). Thirty organoid images were collected for developing OrgaExtractor, and their binary masks were subsequently annotated. Among organoids in various positions, out-of-focus organoids, whose shape could not be recognized owing to phase differences, were excluded from the binary annotation. However, organoids that overlapped with other organoids were separated from each other and included in the binary annotation (Fig. 1b). The images were divided into three groups: 15 for training, 5 for validation, and 10 for testing (Supplementary Table S2).

After setting up the input dataset for development, we established an end-to-end pipeline that analyzes the input image using a DL model^14^. To address the need for hardware-independent environments and balance the trade-off between high computational cost and performance, a multiscale strategy was adopted (Supplementary Fig. S1)^14^. The proposed model produces a binary segmentation output in which black represents the background, and white indicates the organoids.

The basic segmentation is evaluated based on a dice similarity coefficient (DSC) that measures the overlap between the prediction and ground truth (Supplementary Fig. S2). Our model averaged 0.867 for raw outputs of the test set and 0.853 for the post-processed images (Supplementary Fig. S3). The unrefined image could contain true positive pixels that form noisy components, negatively affecting the analysis accuracy. We removed the noisy components for accurate analysis, compromising on DSC. Therefore, we post-processed the raw output using simple image-processing methods, such as morphological transform and contouring. The contour image was considered the final output of OrgaExtractor and was used to analyze organoids numbered in ascending order (Fig. 1c). For a generalizable evaluation, we performed cross-validation with COL-018-N and COL-007-N datasets (Supplementary Fig. S3).

OrgaExtractor provides several measurements, such as the projected area, perimeter, axis length, eccentricity, circularity, roundness, and solidity of each organoid, from the contour image. Although these measurements may be useful for understanding the morphological features of a single organoid, they are insufficient for representing the entire culture condition to which the organoid belongs. Therefore, we added up the measurements, such as the projected area, or averaged out the eccentricity of a single organoid as the parameters of the organoid image to analyze the culture conditions (Fig. 1d). The correlation between analysis parameters and the estimated actual cell numbers was extracted. Based on the most correlated parameter, frequent image analysis enables the estimation of actual cell numbers in a non-invasive manner in real time and provides opportunity to culture organoid samples continuously (Fig. 1e).

### Performance of OrgaExtractor in recognition of organoids comparable to manual recognition

**Figure 2:**
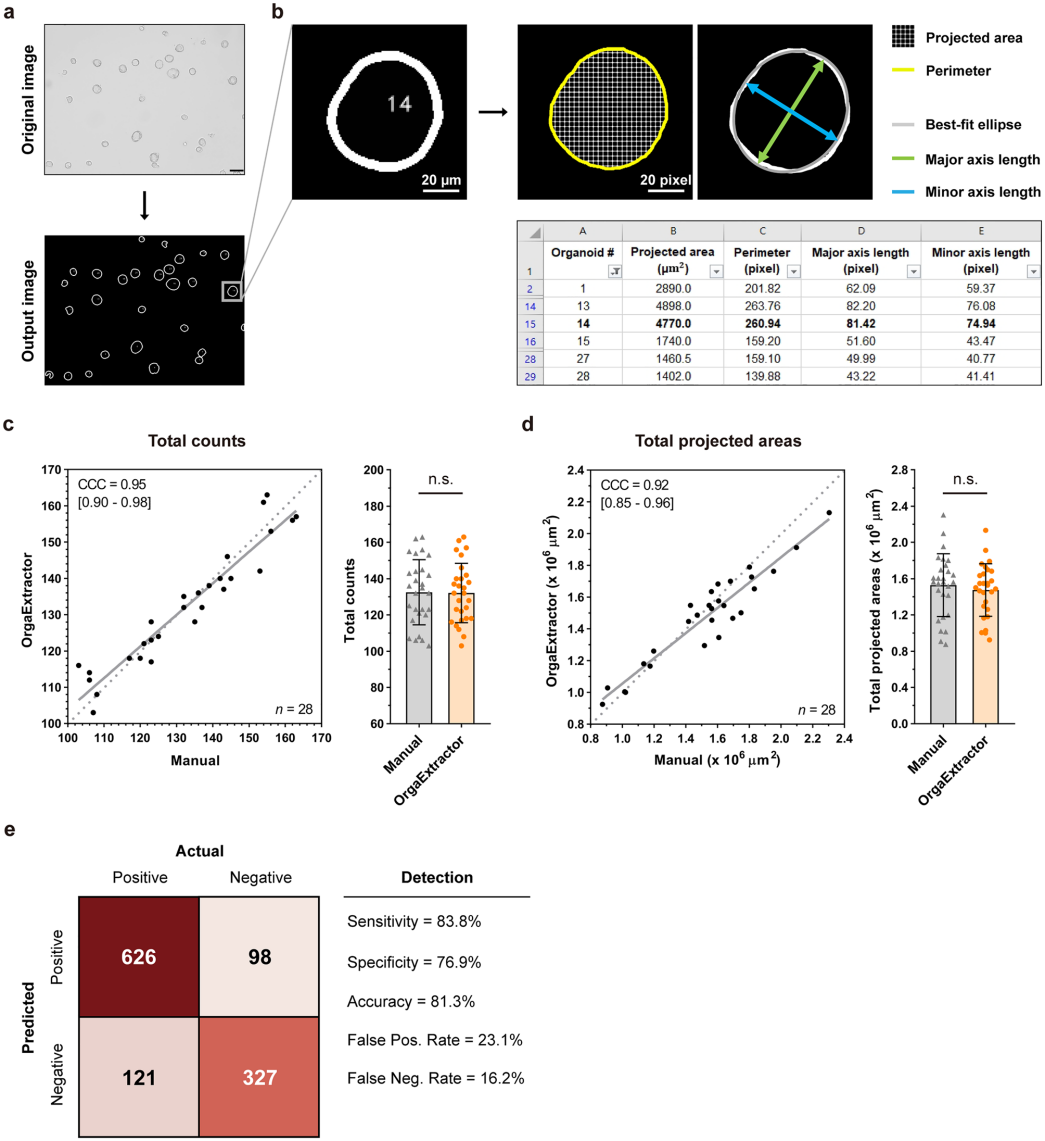
Evaluation of data extracted by OrgaExtractor. (**a**) A pair of organoid images: the original image and its contour image processed by OrgaExtractor (Scale bar = 100 μm). (**b**) Metrics used in this study are visualized, and an excel file of the measurements extracted by OrgaExtractor is shown. See also Supplementary Table S3. (**c**, **d**) The number (**c**) and total projected area (**d**) of counted organoids in 28 images were measured manually and using OrgaExtractor, respectively. Data extracted by OrgaExtractor were compared with manual data based on the concordance correlation coefficient (CCC), exhibiting a 95% confidence interval (CI) (left). Data extracted by OrgaExtractor were compared with manual data and are represented as mean ± SD (right). *n.s.* not significant with a two-sided, independent, two-sample *t*-test. (**e**) Confusion matrix in the context of detection.

The original organoid image was processed using OrgaExtractor, and white organoid contours with black backgrounds were extracted. Each organoid was numbered and marked inside the contours (Fig. 2a). Among the metrics used for the development and evaluation of OrgaExtractor (Supplementary Table S3), the projected area, perimeter, major axis length, and eccentricity were visualized through diagrams. The extracted measurements were saved as a text file in OrgaExtractor, enabling us to handle and manipulate the data efficiently. We calculated the ratio of a micrometer (μm) to a pixel in the original image because the organoid image was saved with a scale bar. The metric projected area (pixels) was converted into the actual projected area (μm^2^) based on the ratio explained (Fig. 2b).

Passaged colon organoids were seeded in a 24-well plate, and 28 colon organoid images were used for quantitative evaluation (Supplementary Table S2). The number and total projected areas of the counted organoids from each image were measured using OrgaExtractor. These data were compared with the corresponding, manually measured data. The number of counted organoids agreed with the concordance correlation coefficient (CCC) of 0.95 [95% confidence interval (CI) 0.90–0.98]. No significant difference between the manual and OrgaExtractor in the total number of counted organoids was observed (Fig. 2c). The total projected areas of counted organoids agreed with the CCC of 0.92 [95% CI 0.85–0.96]. There was no significant difference between the manually measured total projected areas and those measured by OrgaExtractor (Fig. 2d). These results indicate that OrgaExtractor can replace researchers in organoid recognition and measurement. When using DSC, however, simply counting the number of organoids is insufficient because DSC is based on a pixel-by-pixel comparison. Therefore, we used ten pairs of COL-018-N testing datasets to evaluate the performance based on organoid counting. We analyzed our deep learning model with detection methods to observe how many organoids the model can detect. Detection localizes and identifies the presence of organoids recognized by the model, providing the number of organoids that the model finds or misses compared to the ground truth. In the context of detection, OrgaExtractor detects organoids with a sensitivity of 0.838, a specificity of 0.769, and an accuracy of 0.813 (Fig. 2e).

### Organoid measurements from OrgaExtractor correlate with actual culture conditions

**Figure 3:**
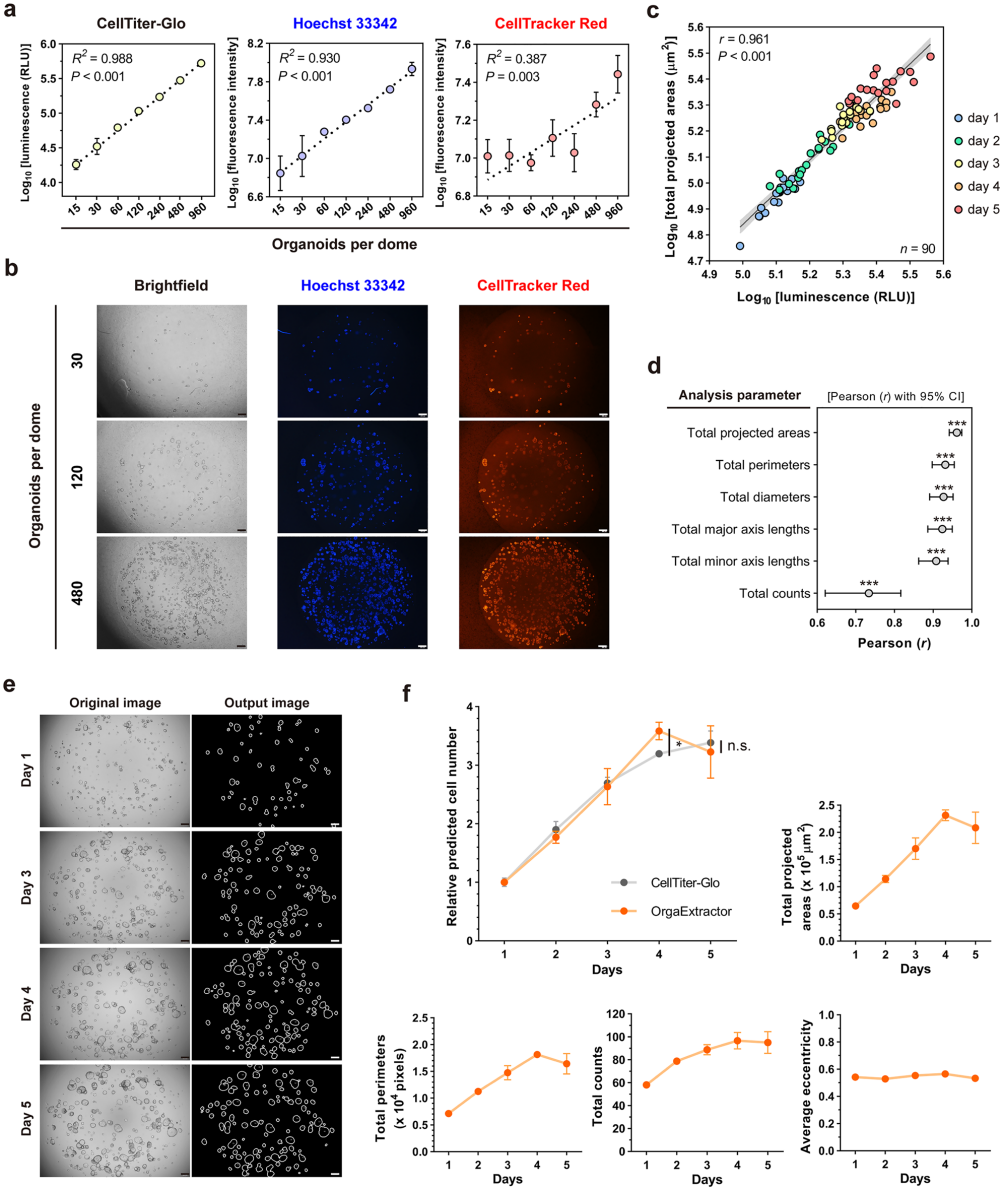
Correlation of data extracted by OrgaExtractor with actual CellTiter-Glo (CTG) assay, Hoechst 33342 staining, and CellTracker Red staining. (**a**) Organoids are seeded with a density of two-fold serial dilution, and their cell numbers are measured with CTG assay, Hoechst 33342 staining, and CellTracker Red staining, shown in triplication (mean ± SD). A coefficient of determination (*R*^2^) with a P-value (*P*) was used to perform a linearization of actual cell numbers. (**b**) Representative brightfield and fluorescence images of organoids, in different seeding densities are shown (Scale bar = 200 μm). (**c**) Organoids imaged on different days are dotted with different colors. The Pearson correlation coefficient (*r*) with a P-value (*P*) was used to compare the total projected areas in the 90 images to the CTG data. (**d**) Other parameters were used to compare the data extracted from the images with the CTG data in Pearson analysis. ***P < 0.001. (**e**) Representative images of 5-day-old, cultured colon organoids (Scale bar = 200 μm). (**f**) Quantitative analysis of organoids are conducted in triplication. Organoid growth was measured using OrgaExtractor (orange) with four different parameters. The total projected areas (orange) were compared with the CTG assay results (gray), and both were plotted as a function of the relative cell number. Data were presented as mean ± SD. *P < 0.05, *n.s.* not significant, using a two-sided independent two-sample *t*-test.

To determine the parameter that can evaluate organoid culture conditions, we first attempted to determine a standard assay for counting actual cell numbers. Organoids under 70 μm in size were seeded in a 96-well plate with a density of two-folded serial dilution. Each well was first stained with Hoechst 33342 and Celltracker Red dye to acquire fluorescence images, followed by the addition of CellTiter-Glo (CTG) reaction solution. Actual cell numbers were relatively estimated using the luminescence values from CTG assay or fluorescence intensities from Hoechst 33342 and CellTracker Red staining assay. Among the assays, the regression predictions of the CTG assay are the best fit to the data (coefficient of determination (*R*^2^) = 0.988, P-value (*P*) < 0.001). Therefore, we used the CTG assay as a criterion for estimating the actual cell numbers in the following experiments (Fig. 3a,b).

Passaged colon organoids under 70 μm in size were seeded in a 96-well plate and cultured for five days. Each well was imaged before the organoid viability was measured using the CTG assay. A total of 90 colon organoid images were used to correlate the data extracted by OrgaExtractor with the actual CTG data (Supplementary Table S2). Pearson analysis revealed a positive correlation (Pearson correlation coefficient (*r*) = 0.961, *P* < 0.001) between the total projected areas of the imaged organoids in each well and the CTG assay results (Fig. 3c). Other parameters related to size were also used to analyze the correlation between the data extracted from the OrgaExtractor and the CTG data. Pearson analysis showed the most robust relationship between the total projected areas and CTG values among other parameters, directly related to size (Fig. 3d). As the luminescence values from the CTG assay scales linearly with the cell numbers, we assumed that the total projected areas of organoids in a single image strongly correlate with the total cell numbers in the same image.

Passaged colon organoids under 70 μm in size were seeded in a 96-well plate and cultured for five days. Three different wells were imaged daily (Supplementary Table S2), before organoid viability was measured using the CTG assay. Representative time-lapse images of the cultured organoids and their output images from OrgaExtractor are shown (Fig. 3e). Data such as total projected areas, total perimeters, total counts, and average eccentricity of 15 images related to Fig. 3e were plotted on each graph. Data of total projected areas from images and CTG assay results from other triplicated wells were both converted to data of predicted cell number, by considering the relative value of one on Day 1, and were plotted on a single graph. Based on the CTG assay results, we empirically found that the growth of cultured organoids has been slowed down on Day 5, which is referred to as the time point for subculture^15^. Triplicated values extracted from the OrgaExtractor were compared with those of the CTG assay results, and no significant difference was observed on Day 5 (Fig. 3f). These findings demonstrate a non-invasive approach to estimating organoid growth, which could potentially be used to determine the optimal time point for subculturing, as illustrated by the use of OrgaExtractor.

### OrgaExtractor enables researchers to understand organoids in the images

**Figure 4:**
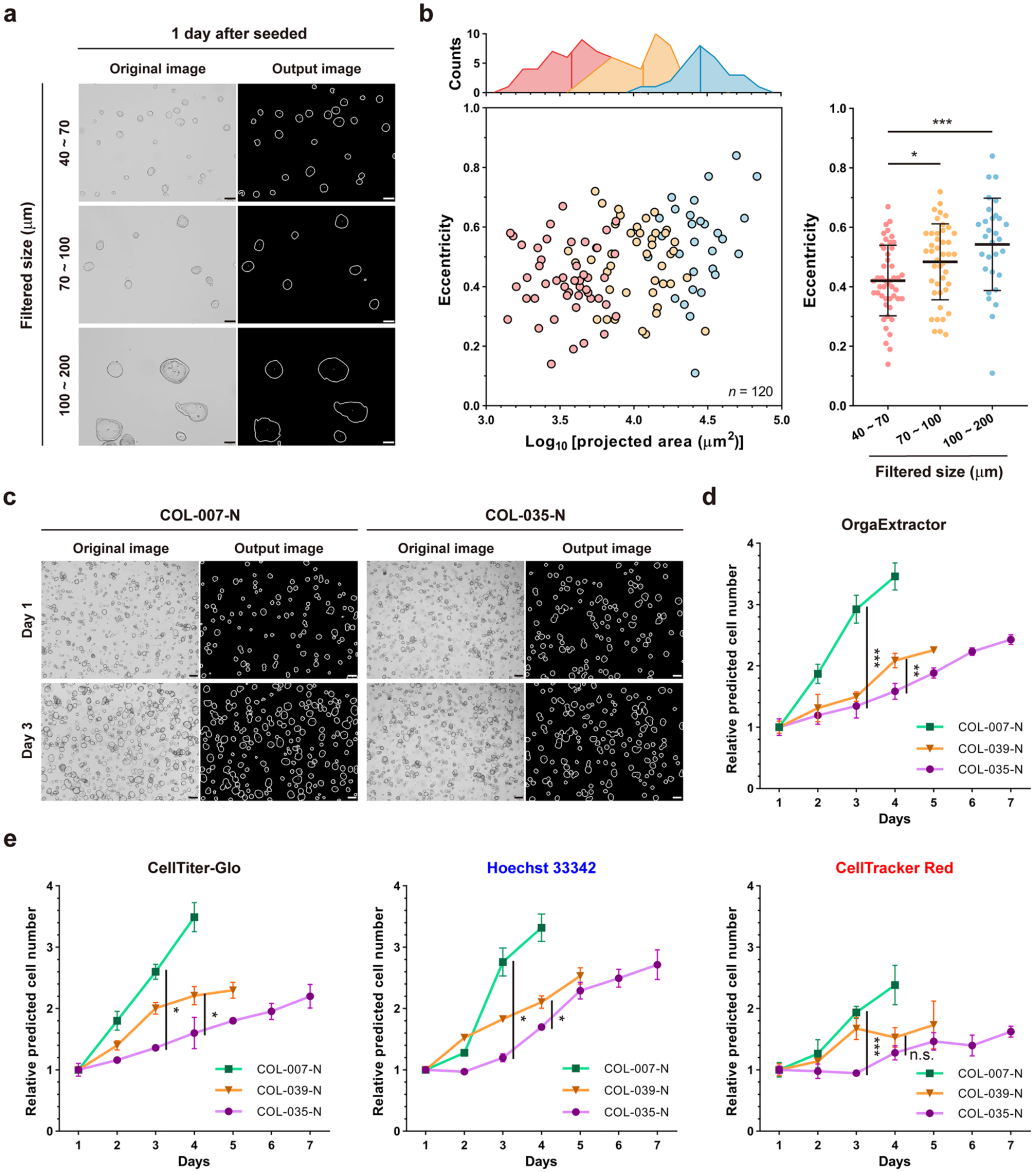
Organoid image analysis with visualization. (**a**) Representative images of differentially filtered organoids (Scale bar = 100 μm). (**b**) The projected area and eccentricity of the organoids are plotted with dots (bottom, left). Organoids are distributed according to the projected area, shown with a mean (top, left) and eccentricity (right). Data are presented as mean ± SD (right). Two-sided independent two-sample *t*-test, *P < 0.05, ***P < 0.001. (**c**) Representative images of two colon organoids, imaged 1 (top) and 3 (bottom) days after seeding in a 24-well plate (Scale bar = 200 μm). (**d**) Data of the total projected areas extracted from three time-lapse images were used to predict relative cell numbers in organoids. Relative cell number was predicted on Day 3 and 4, when COL-007-N and COL-039-N grew faster than the previous day, respectively. Data were presented as means with standard deviations. A two-sided independent two-sample *t*-test yielded **P < 0.01, ***P < 0.001. (**e**) Data of the luminescence value and fluorescence intensity were used to predict relative cell numbers in organoids as a validation of (**d**). Fluorescence intensity from acquired images and luminescence value from the well where the images are captured are normalized, by considering a relative value of one on Day 1. Data were presented as means with standard deviations. A two-sided independent two-sample *t*-test yielded *P < 0.05, ***P < 0.001, and *n.s.* not significant.

Image metrics, such as projected area and perimeter, are directly related to the size of the organoid, regardless of its shape. On the contrary, eccentricity is an image metric that can qualitatively evaluate the shape of each organoid, regardless of its size (Supplementary Table S3). Passaged colon organoids without dissociation were differentially filtered using cell strainers sized 40 μm, 70 μm, and 100 μm. One day after the organoids were seeded in a 24-well plate, 19 images were acquired (Supplementary Table S2). Representative images of organoids in three size ranges, along with the output images, are shown (Fig. 4a). Original images were first processed using OrgaExtractor, followed by the selection of actual organoids. Organoids that were neither cut at the edges nor smaller than 40 μm in size were selected as actual organoids. The projected area and eccentricity of individual organoids measured using OrgaExtractor were plotted on a scatter plot. As organoids were differentially filtered, the data visualized with a marginal plot showed three different distributions in the projected area. We found that the eccentricity of colon organoids filtered between 40 and 70 μm size was smaller than that of other organoids (Fig. 4b).

Three different colon organoids were seeded in a 24-well plate, and 48 images were acquired (Supplementary Table S2). Representative original and output images of different organoids imaged on different days were shown (Fig. 4c). To estimate organoid growth in a non-invasive manner, we analyzed three images per organoid sample using OrgaExtractor and extracted data regarding the total projected areas daily. Based on the data plotted on a graph, organoids were cultured until their growth slowed down. The relatively estimated cell numbers were significantly different between the two rapidly grown organoids and the other gradually grown organoid (Fig. 4d). The growths of COL-007-N, COL-035-N, and COL-039-N were also estimated with CTG assay, Hoechst 33342 staining, and CellTracker Red staining assay, which can also confirm the actual cell numbers (Fig. 4e). As with the Pearson analysis in Fig. 3, image-based data from OrgaExtractor were correlated with luminescence values (CTG assay) or fluorescence values (Hoechst 33342 and CellTracker Red assay) (Supplementary Table S2, Supplementary Fig. S4). These results show that OrgaExtractor can recognize organoids at different stages of growth, and researchers can compare their growth conditions with the data extracted from the images.

## Discussion

Organoids have been widely used as a preclinical model for infectious diseases, cancer, and drug discovery^16^. Since organoids are self-organizing multicellular 3D structures, their morphology and architecture closely resemble the organs from which they were derived^17^. However, these potent features were major obstacles to estimating organoid growth and understanding their cultural condition^18^. Recently, DL-based U-Net models that could detect 2D cells from an image and measure their shape were developed, reducing the workload of researchers^19,20^. In this study, we developed a novel DL-based organoid image processing tool for researchers dealing with organoid morphology and analyzing their culture conditions.

Although MOrgAna and our study fundamentally perform segmentation tasks for organoid images, MOrgAna was trained by a single cropped-out organoid with machine learning and an optional shallow MLP network^12^. Training the OrgaExtractor with a variety of organoids in a single image can result in the extraction of various morphological data. MOrgAna can be widely used in observing a single GFP-expressed organoid in the developmental stage, but OrgaExtractor can be used to estimate the growth of total organoids in a 3D matrix. DeepOrganoid was designed for performing high-throughput viability screens in drug discovery^13^. Although it showed a correlation between the total projected areas of 2D cells and cell viability in the validation stage, understanding the morphology of organoids is necessary. The OrgaExtractor showed a correlation between morphological parameters and organoid viability.

Because U-Net is a prominent convolutional neural network for biomedical image segmentation, we adopted the basic architecture of our model from U-Net^21^. Furthermore, learning the feature maps in multi-scale with a residual path strategy improves the overall model performance of U-Net^14^. With minor modifications, we re-implemented these architectures by including 3 × 3 and 7 × 7 kernels in multi-scale blocks (Supplementary Fig. S1). Therefore, our model accurately segmented the organoid and was able to quantitatively measure each structure in the organoid image (Fig. 1). Furthermore, OrgaExtractor performs segmentation tasks precisely within three seconds per image and provides detailed information regarding each organoid in an image. The ability to analyze a single organoid, even in images containing multiple organoids, allows researchers to avoid capturing each organoid, leading to a more efficient examination process. OrgaExtractor trained with a colon organoid image set can recognize same colon organoid sample from different sets of images with an average DSC of over 0.83 (Supplementary Fig. S3). As OrgaExtractor recognizes organoids in a single image, its organoid measurements are comparable to manual measurements in multiple images (Fig. 2).

Considering the use of image analysis metrics in organoid recognition^22^, we propose that researchers examine total organoids in a single image using parameters that reflect the actual culture conditions of their samples. Various image metrics such as projected area were extracted from every single organoid contour. The total projected areas were then calculated by summing the projected areas of each contour (Fig. 1d). We demonstrated that total projected areas, as an analysis parameter, strongly correlate with the actual cell numbers and can be a parameter for 3D cell counting (Fig. 3c,d). Because the colon organoid in cystic morphology is ellipsoid^15^, the surface area correlated with the projected area is proportional to cell numbers^23^. Considered together, our findings suggest that the analysis of organoid images using OrgaExtractor could serve as a valuable tool for non-invasive cell number estimation (Fig. 3f). Analysis parameters shown in Fig. 3d are derived from image metrics that are inevitably related to the size. Although those parameters can be used for cell number estimation, it is slightly difficult to qualitatively evaluate the morphology of a single organoid. As the morphology of a single organoid can be changed by experimental conditions or stimuli^24^, we attempted to find the morphological features that can be seen during culture. Among the metrics, we characterized the eccentricity of differentially filtered organoids and found that organoids of smaller sizes were less eccentric (Fig. 4b).

The increase in cell number is crucial in both 2D cell line culture and 3D organoid culture^2,16,25–29^. Because 2D cells are maintained as a single cell type, it is relatively easy to count the cell number and anticipate its culture conditions^26,29,30^. However, because an organoid is a multicellular structure of varying sizes, estimating the growth with precise time points is difficult^15,16,30^. OrgaExtractor was used to compare the growth between different colon organoid samples based on the total projected areas and to understand the characteristics of a single colon organoid sample. Researchers can observe the growth of organoid samples in real time using the morphological data extracted from OrgaExtractor. Organoids are heterogeneous in growth (Fig. 4d), and this heterogeneity gives researchers a reason to handle organoid samples individually. Researchers can find suitable culture conditions by subculturing each sample at the optimal time point rather than thoroughly following protocols. Determining the optimal culture conditions for individual organoid samples may prevent unwanted differentiation and expansion termination, followed by a long term maintenance^15^.

There are certain limitations regarding the segmentation of overlapped organoids. Although OrgaExtractor does not recognize blurry out-of-focus organoids that should not be detected, it shows substandard performance on overlapped organoids that are in contact with other organoids. Overlapped organoids have a contact junction that does not appear in a single organoid, making it difficult for OrgaExtractor to segment. The ability to distinguish overlapped organoids as two or more separated organoids is required in future work.

In conclusion, to analyze the morphology and diverse sizes of organoids in images, we developed OrgaExtractor, a DL-based organoid image-processing tool. The data extracted by OrgaExtractor (the parameter used is the total projected areas) correlated with the actual cell numbers in organoids. Researchers unfamiliar with programming can readily use OrgaExtractor to handle images and extract their preferred data. We anticipate that OrgaExtractor will be frequently used at benches, where researchers struggle to optimize the culture conditions of their organoid samples.

## Materials and methods

### Patient specimen collection

All experimental procedures were approved by the Institutional Review Board (IRB) of Yonsei University College of Medicine (Permit Number: 4-2019-0811), and were performed in accordance with relevant guidelines. The study obtained informed consent for specimens from patients who had undergone surgical resection. Patients who underwent surgery to remove cancerous colon tissues contributed healthy colon tissues adjacent to the cancer region (Supplementary Table S1).

### Establishment and culture of normal colon organoids

Tissues adjacent to the cancer region were chemically dissociated using a Gentle Cell Dissociation Reagent (GCDR; #100-0485, Stemcell) for 30 min at room temperature (RT), followed by mechanical dissociation. Pre-chilled Matrigel (#356231, Corning) was added to the media containing isolated organoids as 50% (v/v). After gentle re-suspension, 50 μL of the mixture was dispensed into each well of a 24-well culture plate and polymerized for 30 min at 37 °C. Organoids were cultured in IntestiCult Organoid Growth Medium (OGM; #06010, Stemcell) containing 1X Penicillin/Streptomycin (#15140-122, Gibco) and 10 μM Y-27632 (#Y0503, Sigma-Aldrich) at 37 °C and 5% CO_2_. Media were exchanged every 2–3 days, and organoids were passaged 1:3–1:4 every 5–7 days. For passaging, organoids were dissociated in GCDR for 10 min at RT, followed by mechanical disruption using a P1000 pipette, and finally embedded in diluted Matrigel.

### Cell viability assay

Organoids were dissociated in GCDR for 10 min at RT, followed by mechanical disruption. Small organoids with < 70 μm diameters were collected using a cell strainer (#93070, SPL Life Sciences). After gentle re-suspension with diluted Matrigel, 50–60 organoids per 6 μL were seeded on a white flat-bottom 96-well plate, followed by polymerization for 15 min at 37 °C. Every three days, 200 μL of OGM was added to each well and exchanged. To measure the relative cell number, the medium was removed, and 30 μL of CellTiter-Glo 3D Reagent (#G9682, Promega) was added to each well, followed by vigorous shaking for 20 min at RT. Luminescence, detected using Centro XS^3^ LB 960, was read using MikroWin 2000, and the normalized data were plotted using GraphPad Prism 9 software. A single experiment was performed by triplication.

### Fluorescence staining assay

The methods of seeding the mechanically dissociated organoids for viability assay are aforementioned. After washing each well with phosphate-buffered saline (PBS), 200 μL of PBS containing 7.5 μg/mL Hoechst 33342 (#14533, Sigma-Aldrich) and 10 μM CellTracker Red CMTPX (#C34552, Invitrogen) was added to each well, followed by incubation for 30 min at 37 °C^31,32^. The fluorescence intensity of captured images was quantified using ImageJ^33^, and the normalized data were plotted using GraphPad Prism 9 software. A single experiment was performed by triplication.

### Image acquisition

Images of organoids embedded in Matrigel-containing droplets were acquired using an IX73 inverted microscope (Olympus) with 4 × and 10 × objectives in a brightfield and fluorescence. Because colon organoids were suspended in Matrigel, the level with the most organoids in focus was chosen. The captured images were saved as JPGs (1600 × 1200 pixels) along with a scale bar, using the cellSens standard.

### Manual process of original image into binary mask

In the development process of the OrgaExtractor model, which employs supervised learning, binary masks were generated to correspond with the organoid images. These masks served as ground truths for comparison with the predictions of the DL model. The establisher (T.K.K.) of the patient-derived normal colon organoids visually annotated the regions of projected organoids in the original image. Organoids that were neither cut at the edges nor less than 40 μm in size were outlined. The outlined regions were filled with white, whereas the background was filled with black.

### Datasets

We used the OrgaExtractor development dataset comprising 30 colon organoid (PDO ID: COL-018-N) images. We first acquired 30 in-house original organoid images, and 30 additional binary masks were subsequently annotated by the aforementioned methods. The images in the dataset were split into three groups: 15 for training, 5 for validation, and 10 for testing (Fig. 1).

We used the OrgaExtractor evaluation dataset containing 248 colon organoid (PDO ID: COL-007-N, COL-035-N, and COL-039-N) images. As shown in Fig. 2c and d, 28 images annotated with 28 binary masks were used to evaluate the concordance correlation. As shown in Fig. 3c and d, 90 images were used to evaluate whether the extracted data from the organoids correlated with the actual CTG data. As shown in Fig. 3e and f, 15 images were used to show that the data extracted by OrgaExtractor reflected organoid growth conditions. As shown in Fig. 4a and b, 19 images were used to compare the characteristics of organoids that were different in size. As shown in Fig. 4c and d, 48 images were used to compare the growth rates of different organoid samples. As shown in Fig. 4e, 48 images were used to compare the growth rates of different organoid samples.

The summary information of acquired image datasets is presented in Supplementary Table S2.

### OrgaExtractor model

The detailed model architecture of OrgaExtractor is shown in Supplementary Fig. S1. In the training process, owing to the large original image size and the small size of the dataset, the images were cropped to 512 × 512 and augmented, including random flipping, rotation, affine transformation, and Gaussian blurring. Subsequently, the images were fed into a multi-scale residual U-Net with 3 × 3 and 7 × 7 kernels. We adopted a batch size of eight, a stochastic gradient descent optimizer, and a learning rate of 0.001. Our model is trained based on a compound of dice and cross-entropy loss because a compound loss provides robust results on segmentation tasks^34^. The loss of training and DSC of validation are shown in Supplementary Fig. S3. The related Eq. (1) is as follows:

1 $$L\_total = L\_(CE) + L\_Dice$$

The raw output image from the model is post-processed iteratively with a morphological transformation to remove small components and recover holes. This process was empirically optimized. Finally, OrgaExtractor generates a binary contour image of organoids in which each organoid is labeled in ascending order. It analyzes the contour image using the OpenCV-Python library and provides information such as the projected area, diameter, perimeter, major axis length, minor axis length, eccentricity, circularity, roundness, and solidity.

### Quantification and statistical analysis

All figures were constructed using GraphPad Prism 9 software. All graphs display the mean values (mean) and the error bars represent the standard deviation (SD). Statistical analyses were conducted via the concordance correlation test shown in Fig. 2c and d, linear regression analysis in Fig. 3a, and Pearson’s correlation test shown in Fig. 3c and d, and Supplementary Fig. S4d. P-values were calculated using a two-sided independent two-sample *t*-test in Figs. 2c,d, 3f, 4b,d, and e. *P* < 0.05 was considered statistically significant. *P < 0.05, **P < 0.01, ***P < 0.001.

### Supplementary Information

Supplementary Information.

## Data Availability

The datasets and codes generated and/or analyzed during the current study are available in the GitHub repository: https://github.com/tpark16/orgaextractor.
