# Supplementary material for: Development of a deep learning based image processing tool for enhanced organoid analysis
Source: Sci Rep. 2023 Nov 13;13:19841. doi: 10.1038/s41598-023-46485-2 (PMC10646080; doi:10.1038/s41598-023-46485-2)
Supplement: Supplementary file 1 — Supplementary Information. [file 41598_2023_46485_MOESM1_ESM.pdf]

Development of a deep learning based image processing tool for enhanced organoid analysis

Taeyun Park[1,†], Taeyul K. Kim[2,†], Yoon Dae Han[3], Kyung-A Kim[2], Hwiyoung Kim[4,5,6,*], and Han Sang Kim[2,6,7,*]

[1]Department of Artificial Intelligence, Yonsei University, Seoul, Korea

[2]Department of Internal Medicine, Graduate School of Medical Science, Brain Korea 21 Project, Yonsei University College of Medicine, Seoul, Korea

[3]Department of Surgery, Yonsei University College of Medicine, Seoul, Korea

[4]Department of Biomedical Systems Informatics, Yonsei University College of Medicine, Seoul, Korea

[5]Center for Clinical Imaging Data Science (CCIDS), Yonsei University College of Medicine, Seoul, Korea

[6]Institute for Innovation in Digital Healthcare (IIDH), Yonsei University Health System, Seoul, Korea

[7]Yonsei Cancer Center, Division of Medical Oncology, Department of Internal Medicine, Yonsei University College of Medicine, Seoul, Korea

[†]These authors contributed equally to this work

***Correspondence:**

Hwiyoung Kim, Ph.D.

Department of Biomedical Systems Informatics, Yonsei University College of Medicine,

50 Yonsei-ro, Seodaemun-gu, Seoul 03722, Republic of Korea

Phone: +82-10-9077-4307; E-mail: hykim82@yuhs.ac

Han Sang Kim, M.D., Ph.D. **(Lead Contact)**

Yonsei Cancer Center, Division of Medical Oncology, Yonsei University College of Medicine,

50 Yonsei-ro, Seodaemun-gu, Seoul 03722, Republic of Korea

Phone: +82-10-8973-1312; E-mail: modeerfhs@yuhs.ac

Supplementary Table S1. Characteristics of patient-derived organoids (PDO).

PDO ID	Patient ID	Age (year)	Sex	Specimen Location
COL-007-N	YCC-T144	58	M	Descending colon
COL-018-N	YCC-T174	58	M	Ascending colon
COL-035-N	YCC-T262	47	M	Sigmoid colon
COL-039-N	YCC-T277	79	M	Descending colon

Supplementary Table S2. Summary of two OrgaExtractor image datasets.

PDO ID	Number of images in dataset		Classification	Image characteristics	Image acquisition (Brightfield objective)	Image format
	Development	Evaluation				
COL-007-N		176	28[a], Fig. 2cd; 90, Fig. 3cd; 15, Fig. 3ef; 19, Fig. 4ab; 12, Fig. 4cd; 12, Fig. 4e	24-well plate, Fig. 2; 96-well plate, Fig. 3; 24-well plate, Fig. 4abcd; 96-well plate, Fig. 4e	(4×), Fig. 2, 3, and 4cde; (10×), Fig. 4ab	JPG, original images; TIF, binary mask images
COL-018-N	30		30[b], Fig. 1b, 2e	24-well plate	(4×)	JPG, original images; TIF, binary mask images
COL-035-N		42	21, Fig. 4cd; 21, Fig. 4e	24-well plate, Fig. 4cd; 96-well plate, Fig. 4e	(4×)	JPG, original images
COL-039-N		30	15, Fig. 4cd 15, Fig. 4e	24-well plate, Fig. 4cd; 96-well plate, Fig. 4e	(4×)	JPG, original images
**Total**	**30**	**248**				

Notes: [a]For concordance correlation test related to **Figure 2c–2d**, 28 original images with their binary mask images were used. [b]For the development of OrgaExtractor related to **Figure 1b**, 30 original images with their binary mask images were used. To train, validate, and test OrgaExtractor, 15, 5, and 10 images were used, respectively.

Supplementary Table S3. List of image metrics used for evaluation of OrgaExtractor[1-4].

Metric	Description	Range
Count	Number of segmented regions	≥ 1
Projected area	Number of pixels in the segmented region	≥ 1 pixel
Diameter	Diameter of circle with same area as segmented region	≥ 1 pixel
Perimeter	Distance around boundary of segmented region	≥ 8 pixels
Major axis length	Length of major axis of ellipse that fits a set of 2D points best of segmented region	≥ 1 pixel
Minor axis length	Length of minor axis of ellipse that fits a set of 2D points best of segmented region	≥ 1 pixel
Eccentricity	Ratio of distance between foci of ellipse and major axis length	0 (Circle-like ellipse) to 1 (Line)
Circularity	Ratio of $4\pi \times$ (Projected area) divided by (Perimeter)^2	0 (Non-perfect circle) to 1 (Perfect circle)
Roundness	Ratio of $4 \times$ (Projected area) divided by $\pi \times$ (Major axis length)^2	0 to 1 (Perfect circle insensitive to border)
Solidity	Ratio of projected area divided by convex area	0 (Spindly) to 1 (Solid)

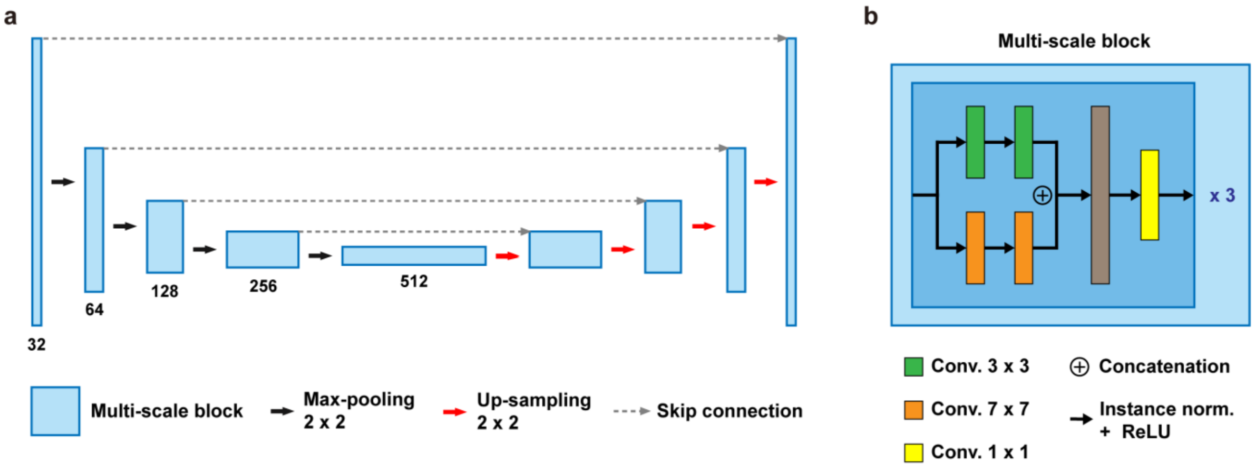

Supplementary Figure S1. Illustration of multi-scale U-Net architecture[5].

(a) Illustration of multi-scale U-Net architecture. **(b)** Multi-scale block includes 3 × 3 and 7 × 7 convolution kernels with instance normalization and ReLU.

a

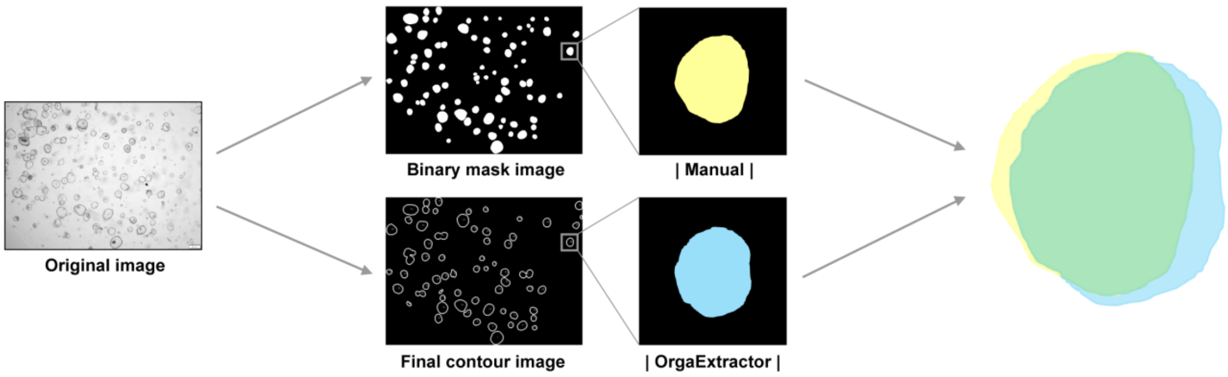

b

$$\textit{Dice Similarity Coefficient (DSC)} = \frac{2 \times |\text{Manual} \cap \text{OrgaExtractor}|}{|\text{Manual}| + |\text{OrgaExtractor}|} = \frac{2 \times TP}{(FN + TP) + (TP + FP)}$$

Supplementary Figure S2. Calculation of Dice Similarity Coefficient (DSC)[6].

(a) Organoids in original image are recognized in manual and by OrgaExtractor, respectively. Two different colors are used to compare the recognized area of the same organoid. Considering the position of organoids in both images, overlapped area of differently recognized organoid is marked in green. **(b)** The equation of DSC. TP, True positive; FN, False negative; FP, False positive.

a

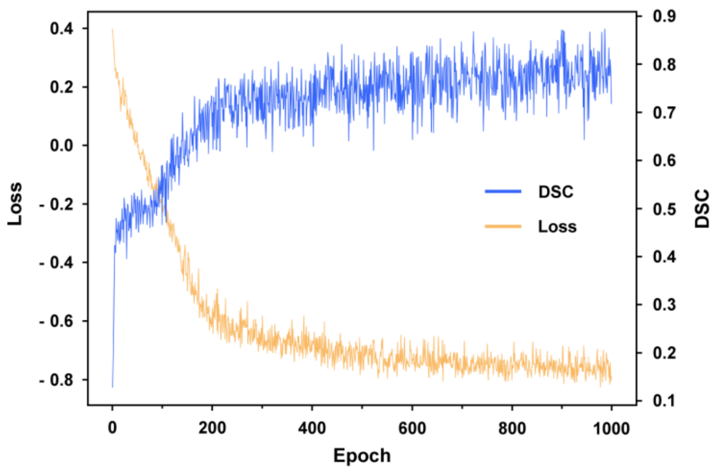

b

Datasets		Average DSC
Training	Testing	
COL-018-N (15 images)	COL-018-N (10 images)	0.853
COL-018-N (15 images)	COL-007-N (28 images)	0.824
COL-007-N (13 images)	COL-007-N (10 images)	0.837
COL-007-N (13 images)	COL-018-N (30 images)	0.744

Supplementary Figure S3. Training and cross-validation of OrgaExtractor[6].

(a) Blue curve indicates DSC of validation, whereas orange curve indicates the loss of training. **(b)** When using the same organoid sample as both training and testing, 30 COL-018-N images are divided into 15, 5, and 10 images, and 28 COL-007-N images are divided into 13, 5, and 10 images to train, validate, and test OrgaExtractor, respectively. Average DSCs of the post-processed images are shown.

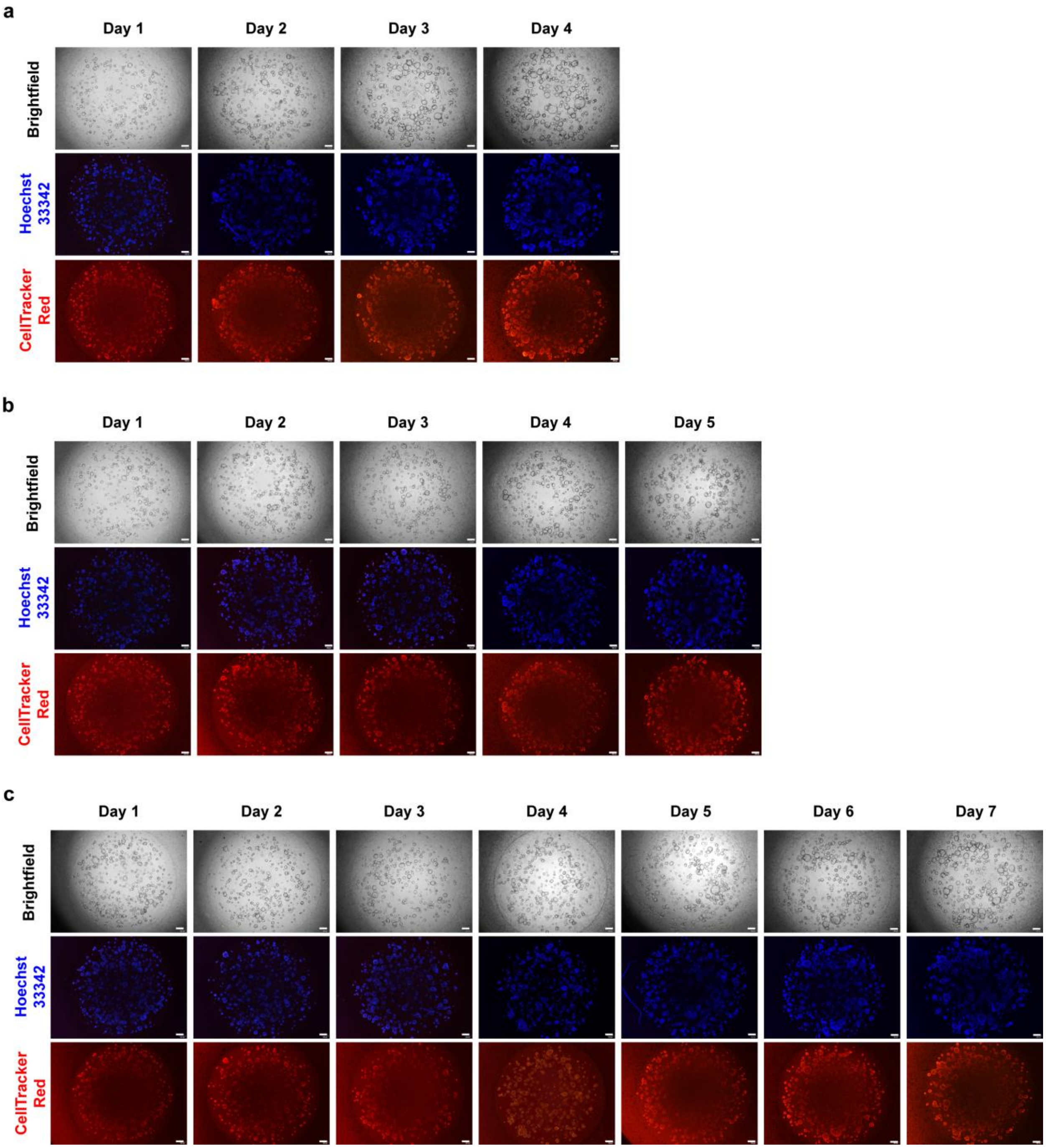
a
Day 1
Day 2
Day 3
Day 4
Brightfield
Hoechst 33342
CellTracker Red
b
Day 1
Day 2
Day 3
Day 4
Day 5
Brightfield
Hoechst 33342
CellTracker Red
c
Day 1
Day 2
Day 3
Day 4
Day 5
Day 6
Day 7
Brightfield
Hoechst 33342
CellTracker Red

d

Analysis parameter	Pearson (r), ***P < 0.001		
	CellTiter-Glo	Hoechst 33342	CellTracker Red
Total projected areas	0.834***	0.708***	0.630***
Total diameters	0.832***	0.669***	0.608***
Total major axis lengths	0.832***	0.672***	0.609***
Total perimeters	0.829***	0.670***	0.607***
Total minor axis lengths	0.822***	0.650***	0.584***
Total counts	0.787***	0.614***	0.585***

Supplementary Figure S4. Validation of OrgaExtractor-based cell number prediction with three different assays

(a), (b), and **(c)** Representative daily brightfield and fluorescence images of COL-007-N, COL-039-N, and COL-035-N are shown, respectively. (Scale bar = 200 μm) **(d)** Analysis parameters from **Figure 3d** were used to compare the OrgaExtractor data of 48 organoid images (**Fig. 4e** dataset) with the CTG, Hoechst 33342, and CellTracker Red data in Pearson analysis, respectively.

Supplementary References

1 Borten, M. A., Bajikar, S. S., Sasaki, N., Clevers, H. & Janes, K. A. Automated brightfield morphometry of 3D organoid populations by OrganoSeg. *Sci Rep* **8**, 5319, doi:10.1038/s41598-017-18815-8 (2018).

2 Pleguezuelos-Manzano, C. *et al.* Establishment and Culture of Human Intestinal Organoids Derived from Adult Stem Cells. *Curr Protoc Immunol* **130**, e106, doi:10.1002/cpim.106 (2020).

3 Takashimizu, Y. & Iiyoshi, M. New parameter of roundness R: circularity corrected by aspect ratio. *Prog Earth Planet Sc* **3**, doi:ARTN 2. 10.1186/s40645-015-0078-x (2016).

4 Zdilla, M. J. *et al.* Circularity, Solidity, Axes of a Best Fit Ellipse, Aspect Ratio, and Roundness of the Foramen Ovale: A Morphometric Analysis With Neurosurgical Considerations. *J Craniofac Surg* **27**, 222-228, doi:10.1097/SCS.0000000000002285 (2016).

5 Su, R., Zhang, D. Y., Liu, J. H. & Cheng, C. D. MSU-Net: Multi-Scale U-Net for 2D Medical Image Segmentation. *Front Genet* **12**, doi:ARTN 639930. 10.3389/fgene.2021.639930 (2021).

6 Isensee, F., Jaeger, P. F., Kohl, S. A. A., Petersen, J. & Maier-Hein, K. H. nnU-Net: a self-configuring method for deep learning-based biomedical image segmentation. *Nature Methods* **18**, 203-+, doi:10.1038/s41592-020-01008-z (2021).
